# Evaluation of a software module for adaptive treatment planning and re-irradiation

**DOI:** 10.1186/s13014-017-0943-4

**Published:** 2017-12-28

**Authors:** Anne Richter, Stefan Weick, Thomas Krieger, Florian Exner, Sonja Kellner, Bülent Polat, Michael Flentje

**Affiliations:** Department of Radiation Oncology, University of Wuerzburg, Josef-Schneider-Str. 11, 97080 Wuerzburg, Germany

**Keywords:** Re-irradiation, Lung cancer, Adaptation, Re-planning

## Abstract

**Background:**

The aim of this work is to validate the Dynamic Planning Module in terms of usability and acceptance in the treatment planning workflow.

**Methods:**

The Dynamic Planning Module was used for decision making whether a plan adaptation was necessary within one course of radiation therapy. The Module was also used for patients scheduled for re-irradiation to estimate the dose in the pretreated region and calculate the accumulated dose to critical organs at risk. During one year, 370 patients were scheduled for plan adaptation or re-irradiation. All patient cases were classified according to their treated body region. For a sub-group of 20 patients treated with RT for lung cancer, the dosimetric effect of plan adaptation during the main treatment course was evaluated in detail. Changes in tumor volume, frequency of re-planning and the time interval between treatment start and plan adaptation were assessed.

**Results:**

The Dynamic Planning Tool was used in 20% of treated patients per year for both approaches nearly equally (42% plan adaptation and 58% re-irradiation). Most cases were assessed for the thoracic body region (51%) followed by pelvis (21%) and head and neck cases (10%). The sub-group evaluation showed that unintended plan adaptation was performed in 38% of the scheduled cases. A median time span between first day of treatment and necessity of adaptation of 17 days (range 4–35 days) was observed. PTV changed by 12 ± 12% on average (maximum change 42%). PTV decreased in 18 of 20 cases due to tumor shrinkage and increased in 2 of 20 cases. Re-planning resulted in a reduction of the mean lung dose of the ipsilateral side in 15 of 20 cases.

**Conclusion:**

The experience of one year showed high acceptance of the Dynamic Planning Module in our department for both physicians and medical physicists. The re-planning can potentially reduce the accumulated dose to the organs at risk and ensure a better target volume coverage. In the re-irradiation situation, the Dynamic Planning Tool was used to consider the pretreatment dose, to adapt the actual treatment schema more specifically and to review the accumulated dose.

## Background

Both plan adaptations and treatments of local recurrence are part of the treatment planning process in daily routine of radiotherapy (RT). Locoregional recurrence is a frequent challenge after primary RT or multimodal treatments. Retreatment approaches of locoregional recurrence depend on disease and patient specific factors (e.g. type and extend of disease, metastatic status, performance status and age of the patient) and pretreatment details (prescription dose, fractionation scheme, target volume size, dose to organs at risk etc.). Additionally, the risk of normal tissue toxicity is a limiting factor in the re-irradiation situation [1]. The necessity of treatment plan adaptation can be caused by changes in body habitus such as weight loss or change of shape or tumor response during the course of treatment.

Adaptive planning is challenging and places substantially burden on the staff. Some commercial vendors are now offering adaptive planning software to stream line the process of re-planning and dose accumulation between different computer tomography (CT) data sets [2]. The Pinnacle Dynamic Planning Module (Philips Radiation Oncology Systems, Version 9.10, Milpitas, CA, USA) provides a tool for treatment plan adaptation and estimation of pretreatment dose for re-irradiation feasible for most clinical situations from a technical point of view. The module provides the user with the tools needed to make decisions and act upon them based on patient changes over time – either during one treatment course or for re-treatments.

The aim of this work is to validate the Dynamic Planning Module in terms of usability and acceptance in the treatment planning workflow. The frequency of using the module for plan adaptation and re-irradiation was assessed depending on the treated body region.

## Methods

### Patient characteristics

The Dynamic Planning Module was evaluated in a time span of one year. This module was used for plan adaptation within one course of radiation therapy. Additionally it was utilized to estimate the dose in the pretreated region and calculate the accumulated dose to critical organs at risk for patients scheduled for re-irradiation in our institution. During one year, 370 patients were scheduled for plan adaptation or re-irradiation (observation time 09/2015–08/2016). All patient cases were classified according to the treated body region: cranial, head and neck, thoracic, abdominal, pelvic or other region (Table 1).

**Table 1:** Overview of scheduled cases for dynamic planning sorted by location and purpose (adaptation or re-irradiation)

Location	Cases		Plan adaptation		Re-irradiation	
	Absolute	Relative in %	Absolute	Relative in %	Absolute	Relative in %
Cranial	29	8	6	4	23	11
Head and neck	36	10	12	8	24	11
Thorax	189	51	97	62	92	43
Abdomen	27	7	16	10	11	5
Pelvis	79	21	21	13	58	27
Other	10	3	4	3	6	3
	370	100	156	100	214	100

Case examples for re-planning will be presented for some treated body regions. Table 2 provides an overview of example cases including treatment concept, fractionation scheme and imaging interval.

**Table 2:** Example cases and treatment concepts (I: main course, II: boost course), imaging interval and delivered fractions before plan adaptation

Case	Disease Type	Treatment concept		Fractionation Scheme (SIB)	Planned fractions	Imaging interval	Delivered fractions
						in days	
#1	Tongue cancer	Primary RCT	I	56.1/66.0/70.95Gy	33	49	6
#2	CUP	Adjuvant RT	I	59.4/66.0Gy	33	48	26
#3	Lung cancer	Primary RCT	I	50.4/61.6Gy	28	12	1
#4	Lung cancer	Primary RCT	I	50.4/61.6Gy	28	32	14
#5	Prostate cancer	Adjuvant RT and pelvic LN	I	45.9/56.7Gy	27	18	1
			II	10.2/12.6Gy	6		
#6	Prostate cancer	Palliative RT	I	40.0/50.0Gy	20	25	8
			II	10Gy	5		

*RCT* Radio-Chemo Therapy, *RT* Radiotherapy, *LN* Lymph nodes, *CUP* Cancer of unknown primacy

### Dynamic planning module

Dynamic Planning provides an automated workflow which includes image fusion, transfer of plan data to a new image set and contours propagation. Fast assessment and automated re-planning tools generate information to help monitor treatment efficacy and create new plans with limited user intervention. Dynamic Planning propagates and optionally deforms contours from an original CT image set that was used for treatment planning to a new image set. Further, records of prior treatment plans are created with read only views of data that include dosimetric evaluation tools. Organ motion and volumes changes can be assessed across treatments including trending for structure volumes, locations and dose. Rigid dose accumulation is feasible for multiple trials and records associated with the primary image set in Pinnacle Version 9.10. Dose deformation is not supported in Pinnacle Version 9.10.

### Dynamic planning workflow

**Fig. 1:**
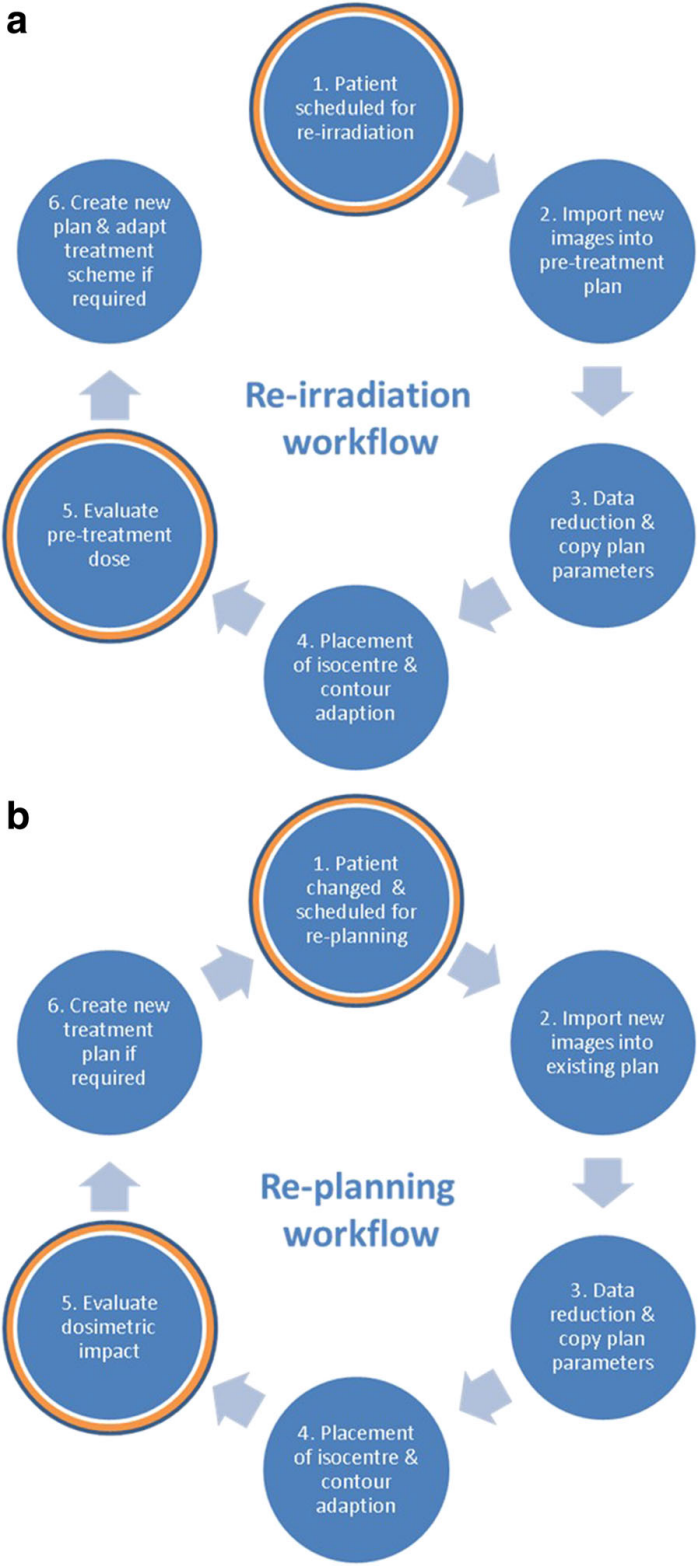
Workflow for the re-irradiation (**a**) and re-planning situation (**b**). Work steps for decision making are highlighted in red

A workflow was developed which is focused on clarity and practicability in clinical routine (Fig. 1). Work steps were defined with responsibilities for the radiation oncologists (work step 1, 4 and 5) and physicists (work step 2, 3 and 6).

The workflow starts with a request of a radiation oncologist for either a plan adaptation during an actual treatment course or re-irradiation of a patient (Fig. 1, Step 1) followed by CT imaging (Fig. 1, Step 2). The tool was involved in the decision for re-treatments if an overlap between pre-treated and actual volume is expected. In this situation, the patient case was scheduled for Dynamic Planning to consider the dose of the pretreatment (Fig. 1a, Step 1) and to evaluate the accumulated dose to the normal tissue and critical organs at risk like the spinal cord (Fig. 1a, Step 5 and 6). Plan adaptation was requested, if any anatomical changes were detected in cone beam CT (CBCT) images during the treatment course. The radiation oncologist was informed and the patient was scheduled for plan adaptation (Fig. 1b, Step 1). For some indications (e.g. in lung cancer) a repeated CT imaging is performed routinely.

For plan adaptation, the repeated CT scan was acquired under the same specifications as in the initial planning CT. In case of re-irradiation, CT image acquisitioning and patient positioning was performed based on the requirements for the actual location (e.g. different patient positioning between pretreatment and actual treatment course). As an assumption for automatic image registration, the same patient positioning (including immobilization devices) should be used when it is adequate for the current treatment.

Before the initial treatment plan was copied to the new image set (Fig. 1, Step 3), the data in the treatment plan was reduced to relevant objects (isocenter, target volumes and organs at risk). This was achieved by removing unnecessary secondary image sets and contours (help contours, points, not applied planning trials, IMRT objectives and AutoPlanning goals). Records of prior treatment plans are created with read only views of data. Once objects appear in the record viewer (contours, points, beams) they cannot be removed anymore. The data reduction was done to enhance the clarity in the trial and to keep the system utilization moderate regarding loading, optimization and dose computation.

Then all plan parameters (image set, points, contours and beams) were copied to the new image data set followed by automatic rigid image registration of the original and new image data set. The option of an automatic contour deformation is principally available in the module, but for the current evaluation, it was not used because of the inverse consistency and transitivity errors in deformable image registration [3, 4]. The image fusion was proofed based on relevant anatomical structures near the target volume or critical organs at risk. The isocenter was positioned in the new image data set based on the initial isocenter position by a radiation oncologist (Fig. 1, Step 4). Target volumes and organs at risk were adapted manually by the radiation oncologist in the new image sets.

Based on the previous steps in the workflow, the dose distribution of the original treatment plan was calculated on the new CT data set with fixed monitor unit setting (Step 5). The next step in case of plan adaptation was to examine whether re-planning was necessary to improve poor target coverage or sparing of organ at risk sparing (Fig. 1b, Step 6). In the re-irradiation scenario, decision making was performed by a radiation oncologist based on the dose distribution of the original treatment plan on the new planning CT (Fig. 1, Step 6). Here, the radiation oncologist takes into account the elapsed time since re-irradiation, pretreatment details (fractionation scheme, target volume size, totally applied dose to target volumes and organs at risk) as well as disease and patient specific factors.

### Sub-group evaluation

For all scheduled patients for plan adaptation, it was assessed how often plan adaptation was required. Plan adaptation during the main and boost course were defined as unintended and intended adaptation, respectively. For 20 patients treated with RT for lung cancer, the dosimetric effect of plan adaptation during the main treatment course was evaluated in detail. This sub-group consists of 20 patients: 18 patients with non-small cell lung cancer (NSCLC), 2 patients with small cell lung cancer. Changes in tumor volume and the time span between treatment start and plan adaptation were recorded. The remaining lung tissue without planning target volume (PTV) overlap was considered for calculation of the mean lung dose. The mean lung dose is well correlated with the risk of radiation pneumonitis as an important dose-limiting toxicity [5, 6] .

## Results

During the first year after implementation, the Dynamic Planning Module was used in 370 cases: 156 for plan adaptation and 214 in consideration of pretreatments for re-irradiation as shown in Table 1. In general, most cases were assessed for the thoracic body region (51%) followed by pelvis (21%) and head and neck cases (10%).

Treatment plans were adapted in 40% during the main treatment course while 36% were adapted for the boost course. In 24% it was decided by the radiation oncologist that no re-planning was necessary.

The main reason for scheduling patients for plan adaptation was anatomical changes which were detected in CBCT images for setup verification: e.g. tumor progression or shrinkage, pleural effusions, edema or organ filling. These changes can influence target coverage or sparing of organs at risk. Another reason for plan adaptation was a change of the positioning devices. Vacuum cushions were renewed due to a leakage in 4% of all cases followed by imaging. The dose distribution was recalculated based on the new planning CT to decide whether re-planning is necessary (Fig. 1b, Step 5 and 6).

### Sub-group evaluation

Out of 156 cases, 53 were scheduled for plan adaptation during RT treatment of lung cancer. Treatment plans were adapted for 20 of 53 cases during the main treatment course which were the subject of a detailed evaluation. An initially intended plan adaptation for the boost treatment course was performed for 23 of 53 cases. No plan adaptation was performed in 10 of 53 cases because no relevant changes of the PTV were observed.

**Fig. 2:**
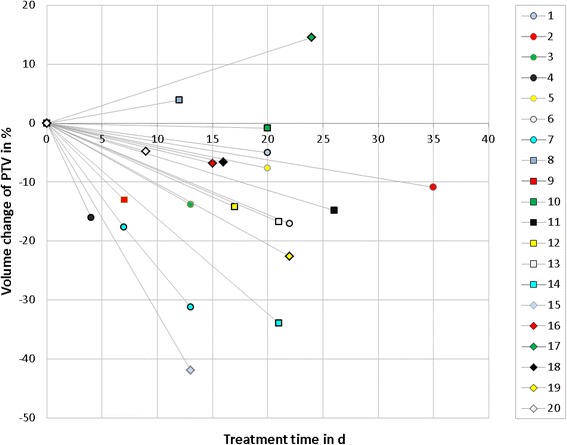
Volume change of PTV as a function of treatment time for 20 patients. The treatment time shows the day within the course at which a request for plan adaptation was scheduled

Figure 2 shows the relative change of PTV as a function of treatment time. Time span between first day of treatment and necessity of adaptation was 17 days in median, range 4–35 days. A relative change of PTV was 12 ± 12% on average (maximum change 42%). In absolute volume, PTV changed by 140 cm^3^ on average (maximum 487 cm^3^). PTV was reduced in 18 of 20 cases due to tumor shrinkage and enlarged in 2 of 20 cases. An increase in PTV of 162 cm^3^ (4%) was seen in case #17 because of a growing pleural effusion. In this case the PTV was modified according to the deformed anatomy in the planning CT followed by a treatment plan adaptation. The new treatment plan resulted in a higher mean lung dose. For case #8 the PTV was enlarged by 15% (49 cm^3^) due to an increasing ventilation disorder during the treatment course.

**Fig. 3:**
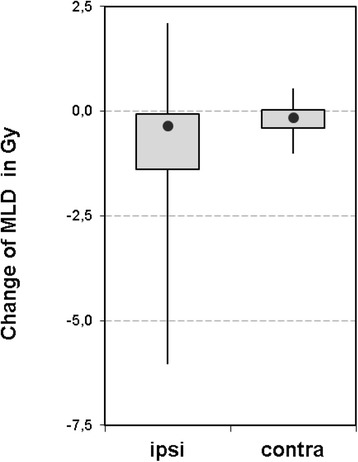
Boxplot showing the change of mean lung dose after plan adaptation. For the subgroup of thoracic cases, mean dose was assessed for the ipsilateral and contralateral side of the lung. The box displays the upper and lower quartile and the median value (filled circle). The whiskers illustrate the minimum and maximum values

For the sub-group of 20 patients, the dosimetric effect of PTV modification followed by plan adaptation was evaluated. Figure 3 shows the change of mean dose for the ipsilateral and contralateral side of the lung. In total, the dose of the ipsilateral lung changed by −0,4 Gy in median (range − 6 - 2,1 Gy). In 15 of 20 cases the mean dose of the ipsilateral lung decreased by 0,6 Gy in median (range 0,1–6 Gy). In 5 of 20 cases the mean dose of the ipsilateral lung increased by 0,7 Gy in median (range 0,1–2,1 Gy).

### Example cases

**Fig. 4:**
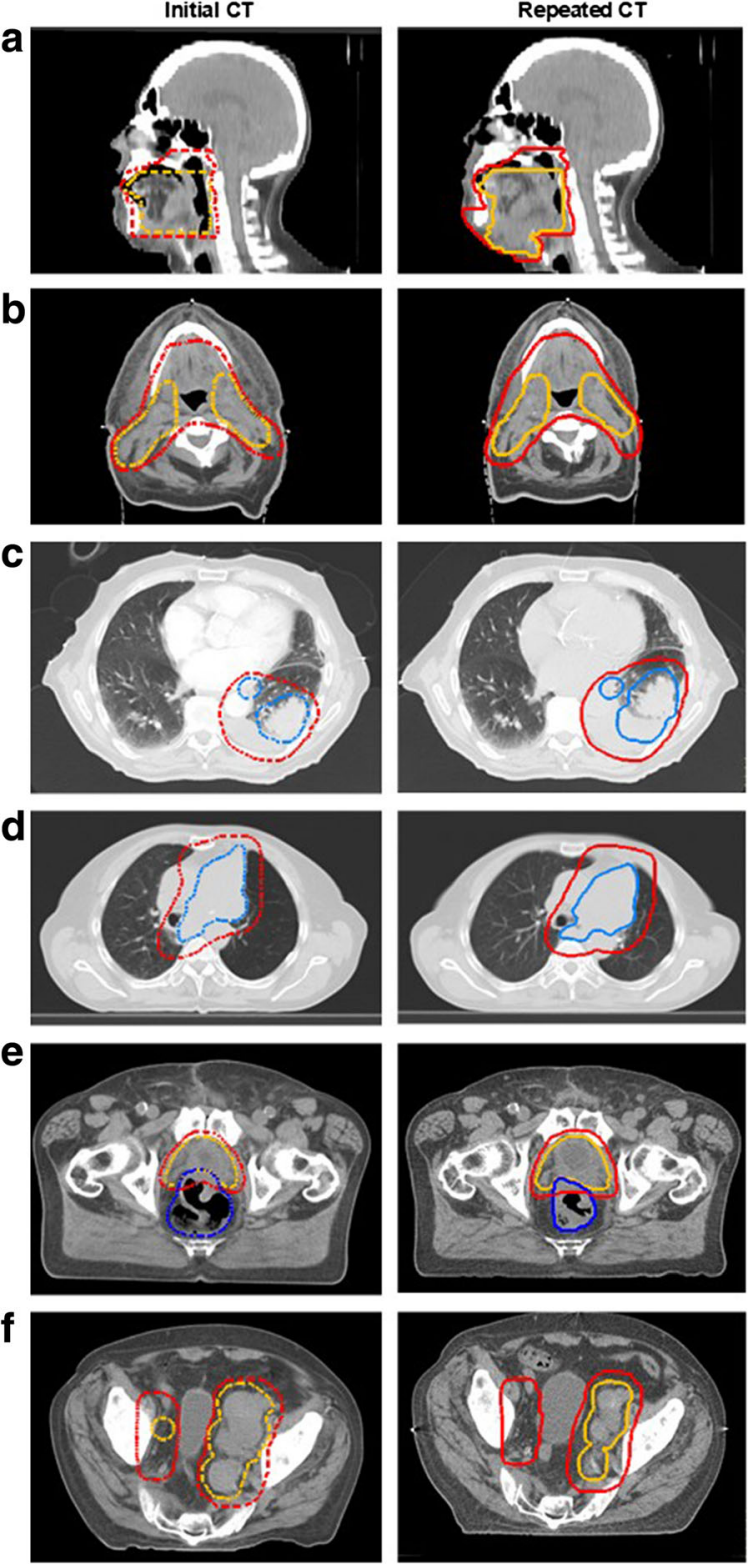
Example cases of anatomical changes during the treatment course for different entities. PTVs are shown in the initial CT (dashed lines, left side) and adapted PTVs in the repeated CT (solid lines, right side): **a** Head and neck case of tongue cancer (left) with tumor progress (right). The PTVs receiving 56,1 Gy and 66 Gy are highlighted in red and orange, respectively. **b** Head and neck case and CUP syndrome (left) with volume change due to edema reduction (right). PTVs receiving 59,4 Gy and 66 Gy contours are highlighted in red and orange, respectively. **c** Thorax case with lung cancer (left) and a growing pleural effusion (right). PTV receiving 50,4 Gy and 61,6 Gy are highlighted in red and blue, respectively. **d** Thorax case with lung cancer (left) and a mediastinal shift (right). PTVs receiving 50,4 Gy and 61,6 Gy are highlighted in red and blue, respectively. **e** Pelvis case with prostate cancer (left) and change in rectal volume (right). PTVs receiving 45,9 Gy and 56,7 Gy are highlighted in red and orange, respectively. **f** Pelvis case with prostate cancer and irradiation of bone and lymphatic metastasis (left) and decrease in lymph node size (right). PTVs receiving 40 Gy and 50 Gy are highlighted in red and orange, respectively

Example cases for several entities are shown in Fig. 4 for the re-planning situation. Anatomical changes during the treatment course were found resulting in a PTV modification and plan adaptation. Table 2 lists imaging intervals and number of delivered fractions before plan adaptation.

A case of tongue cancer is depicted in Fig. 4a (case #1). The dose was prescribed to three dose levels 56.1/66.0/70.95Gy (33 fractions). After 6 delivered fractions, anatomical changes were detected due to edema and the patient was scheduled for plan adaptation. CT imaging was repeated after 49 days and 6 delivered fractions. PTV was enlarged from 1395 cm^3^ to 1589 cm^3^ followed by re-planning to ensure a better target coverage.

Figure 4b shows an example case #2 for a cancer of unknown primacy in the head and neck region. A simultaneous integrated boost (SIB) technique was applied to deliver 59.4 Gy and 66 Gy to the PTV1 and PTV2. A decreased edema was observed during the treatment course. Imaging was repeated after 26 delivered fractions (imaging interval 48 days). PTV was modified from 1627 cm^3^ to 1314 cm^3^.

Example case #3 is demonstrated in Fig. 4c. The patient with lung cancer was treated with a SIB technique delivering 50,4 Gy and 61.6 Gy (28 fractions) to the mediastinal lymphatics and macroscopic tumor. During setup verification of the first treatment fraction, a growing pleural effusion was detected in the CBCT. The patient was scheduled for plan adaptation. Imaging interval was 12 days. PTV changed by 162 cm^3^ (from 1113 cm^3^ to 1275 cm^3^).

Example case #4 is illustrated in Fig. 4d. The patient was treated with a SIB technique as in case #3. After 32 days and 14 delivered fractions, a mediastinal shift was observed. The gross tumor volume (GTV) moved by 1,3 cm, 0,7 cm and 1 cm in lateral, vertical and longitudinal direction, respectively. PTV and GTV changed by 68 cm^3^ and 63 cm^3^ respectively and resulted in re-planning.

Example case #5 is shown in Fig. 4e. The patient was adjuvantly treated after radical prostatectomy with a SIB technique delivering 56,7 Gy to the prostate bed while simultaneously delivering 45,9 Gy to the pelvic lymph nodes in 27 fractions. A sequential boost course followed delivering 10,2 Gy and 12,6 Gy (6 fractions) to the seminal vesicles and to the prostate bed, respectively. During treatment the rectal filling varied persistently and resulted in a PTV modification and re-planning.

A second case with pelvic irradiation is shown in Fig. 4f (case #6). A patient with lymphatic and bone metastasis from prostate cancer was treated with a SIB technique to deliver 40 and 50 Gy (20 fractions) for pain control and lymph edema. An impressive tumor regression was observed in the lymph nodes after 8 fractions. CT imaging was repeated and the PTVs for the SIB technique were reduced by 190 cm^3^ and 160 cm^3^ followed by re-planning.

### Limitations

For re-irradiation, difficulties in image registration occurred when the immobilization techniques or positioning devices differed between the CT scans. Table 3 gives an overview of occurred failures during automatic image registration. In 8% of the scheduled cases image registration was not optimal due to different arm positioning. For some entities (e.g. in lung cancer) the arms up position is required to avoid passing beams through the arms. In contrast other entities (cranial or head neck) required an arm down positioning. For these cases, a manual image registration was performed by the radiation oncologist based on the relevant anatomical structures.

**Table 3:** Overview of failures during automatic image registration

	Re-irradiation	
Limitation	Absolute	Relative in %
Arm position	18	8
Head or pelvis rotation	4	2
Patient position	2	1
Diaphragm control	1	0,5
	26	12

The workflow failed in 1% of all re-irradiation cases when patient orientation during imaging differed between initial and current CT (e.g. head first, feet first, prone or supine). The workaround procedure was to export and import of treatment plans (beams and points) into the new image set. The beam configuration was adapted to the new patient position by changing gantry and collimator followed by a re-calculation of dose.

If changes in anatomy were observed, the initial isocenter was placed approximately based on landmarks in the new image. Another approach would be to use dose deformation based on deformation maps from non-rigid image registration which is not available in the Pinnacle Version 9.10.

In addition, the usual workflow was interrupted if the original plan contains a bolus contour. In this case (10% of scheduled cases) the bolus contour was created as an additional region of interest with a density override.

Despite some limitations for re-irradiation, the workflow for re-irradiation or plan adaptation was successfully applied for each scheduled case. Cases with limitations were handled by using work arounds (e.g. different arm position, bolus contour or CT orientation).

## Discussion

The experience showed high acceptance of the Dynamic Planning Module in our department for both physicians and medical physicists. It was used in 20% of treated patients per year (370 of approximately 1800 patients). The Dynamic Planning procedure was requested for both approaches nearly equally (42% plan adaptation and 58% re-irradiation). It was mostly used for thoracic, head and neck and pelvis cases. Especially for retreatments it was a useful tool to consider dose of the pretreatment and to evaluate the accumulated dose to the normal tissue and critical organs at risk like the spinal cord. For plan adaptation, it assisted the radiation oncologist to decide that plan adaptation was necessary in 76% of all scheduled cases. In addition, the Dynamic Planning Module was used after treatment course interruptions for verification of target coverage in the original treatment plan on the latter CT.

The relevance of retreatments for several indications was described by Mantel et al. in a review focused on stereotactic re-irradiation [5]. They stated that loco-regional relapse is frequent after definitive RT or multimodal treatment. Re-irradiation in the head and neck, thoracic and pelvic region has gained interest with availability of SBRT and preliminary clinical results are promising [5]. This is in agreement with the observation in the current work, that Dynamic Planning was mainly requested for thorax, head and neck and pelvis cases. This supports the need of a reliable software tool with a good usability for dose estimation in the re-irradiation situation.

Beside Dynamic Planning, other vendors provide tools for deformable image registration, dose tracking and decision making. Different deformation algorithms are implemented in the tools and misregistrations can influence the accuracy of dose tracking and dose accumulation. The performance of algorithms for deformable image registration was validated based on phantom studies [7, 8]. Pukala et al. performed benchmarking of five commercial DIR algorithms (MIM, Velocity, RayStation, Pinnacle and Eclipse) using a set of 10 virtual phantoms [8]. Misregistrations were encountered which produced mean and max errors up to 6.8 mm and 22 mm. It was suggested, that strategies which include DIR uncertainty into the dose adaptive process should be considered. Two adaptive planning tools (Pinnacle Dynamic Planning and MIM software) were compared by Stanford et al. with dose differences of 6–9% [2]. The deviation was due to the different ways of dose estimation. The MIM software deformed the dose distribution based on the deformation map between the image sets, whereas Pinnacle re-calculated the dose distribution on the new image set. Pinnacle considers geometrical changes and the effect of radiological path length on dose distribution.

Another approach for cumulative dose assessment (named SCUDA) was developed by Park et al. [9]. Additionally, an overview of seven adaptive RT systems was given with available functionalities: CT-CBCT registration, dose computation and accumulation, automatic segmentation and data retrieval. Park et al. stated that RayStation (Raysearch Laboratories, Sweden) and SCUDA are equipped with all functionalities. Further, SCUDA is optimized for CT-CBCT registration and allows necessary modifications to connect multiple data platforms in the clinical workflow. Pinnacle also provides modules with all integrated functions but the workflow still requires user intervention and is not fully automated in its current form.

Before using the Dynamic Planning Module, the workflow was based on a manual simulation of all steps in the workflow (Fig. 1). In case of re-irradiation, the procedure was to export and import of treatment plans (beams, points) into the new image set. Contours were copied to the new image data set after image registration. In case of plan adaptation, the new image set was added and registered as secondary image set. The visual comparison of both image sets by a radiation oncologist led to the decision if re-planning was necessary or not. Dose calculation was limited to the primary (initial) CT. Altogether, the manual procedure was very time consuming with an intensive workload and was only used in specific cases.

Treatment plan adaptation in the re-planning situation was evaluated for head and neck cases by several groups [10–12]. The aim was to determine re-planning criteria and timing for plan adaptation. Yoa et al. found valuable indicators (initial parotid volume, initial parotid mean dose, and weight loss rate) for parotid protection-based re-planning for nasopharyngeal cancer [12]. Wang et al. stated that re-planning before the 25th fraction during IMRT helps to ensure adequate dose coverage to the target volumes and safe doses to critical normal structures [11]. Castadot et al. reviewed techniques of adaptive RT for head and neck tumors and discussed their potential for maximization of therapeutic index [10]. Another overview was given by Brouwer et al. about anatomic changes of head and neck organs at risk and dosimetric consequences during RT to identify patients who may benefit from adaptive RT and find potential pre-treatment selection criteria [13]. Both review articles concluded that there is a need for larger and well-designed and well-powered prospective studies and practical guidelines to address the safety and the clinical effect of re-planning on patient outcome [10, 13].

Benefits of adaptive RT in lung cancer were investigated using an isotoxic criteria on the mean lung dose and dose escalation in the target volume in several studies [14–16]. Different adaptation schemes were compared to determine the best timing for adaptation. Guckenberger et al. observed a dose escalation of 6 Gy and increased TCP by 9% on average when re-planning is performed in weeks 3 and 5 during the treatment course [15]. In another study an average increase of 13.4 Gy in the target volume was achieved for adaptation in weeks 2 and 4 [16]. In a further study, synthetic image sets were utilized to simulate different adaptation schedules with the objective to characterize the tradeoff between the adaptive benefit and re-planning frequency [14]. Dial et al. summarized that the majority of benefits was obtained with a single mid treatment adaptation. Further, the workload associated with daily adaptation outweighs additional benefit. However, as the workload associated with planning decreases through the development of automatic methods, daily re-planning may be justified.

Re-planning strategies for pelvic tumours were summarized in a review by Thörnqvist et al. [17]. They collected data about type and frequency of imaging to identify anatomical variations, the type of decision criteria used to trigger adaptations, any dose prescription modifications. Various adaptation strategies are in development which were classified by Thörnquist et al. into online or offline strategies and depending on the type of modification executed (e.g. plan selection or re-planning/re-optimization). It was stated that adaptation was dominated by offline re-planning or re-optimization [17]. An offline- re-planning strategy was presented by Bratengeier et al. The technique adapts step and shoot intensity modulated radiotherapy plans by modifying only the segment shapes and preserving the monitor units [18, 19]. Another approach would be to generate motion robust treatment plans that are robust against interfraction and intrafraction motion [20, 21]. Online workflows have increased with the availability of in-room imaging systems like CT or MRI. As already mentioned for head and neck and lung cancer, it was summarized by Thörnqvist et al. that clinical implementation of adaptive RT is rare due to challenges of re-contouring and patient selection [17].

As a technological innovation, adaptive RT may become a new treatment standard and eventually replaces the classical treatment plan in the routine clinical practice. However, adaptive RT remains extremely time-consuming and automation of the different processes involved will be required. Beside the technical automation, prospective studies and practical guidelines are needed to identify patients that may benefit from the adaptive RT and find predictive parameters for re-planning.

## Conclusion

A high acceptance of the Dynamic Planning Module was observed in our department for both physicians and medical physicists. For this acceptance, the development of an optimized workflow was essential. The Dynamic Planning Tool was used for both plan adaptation and re-irradiation in nearly similar parts - mostly for thoracic, head and neck and pelvic region. The evaluation showed that re-planning was necessary in 76%. The re-planning can potentially reduce the accumulated dose to the organs at risk and ensure a better target volume coverage.

In the re-irradiation situation, the Dynamic Planning Tool was used to estimate pretreatment dose. According to the tolerance dose of the individual organs at risk and relapsed time since irradiation, the radiation oncologist can adapt the actual treatment schema more specifically. Consequently, the pretreatment dose can be considered during treatment plan optimization. The total dose distribution can then be reviewed by accumulating the pretreatment and actual dose.

## Abbreviations

CBCT: Cone beam computer tomography
CT: Computer tomography
CUP: Cancer of unknown primacy
GTV: Gross tumor volume
LN: Lymph node
NSCLC: Non small cell lung cancer
PTV: Planning target volume
RCT: Radio chemo therapy
RT: Radiotherapy
SCUDA: Software platform for cumulative dose assessment
SIB: Simultaneous integrated boost

## References

[1] Mantel F, Flentje M, Guckenberger M (2013). Stereotactic body radiation therapy in the re-irradiation situation--a review. Radiat Oncol.

[2] AAPM Meeting in Anaheim, Canada in the Journal Medical Physics (The International Journal of Medical Physics Research and Practice) Med Phys. 2015;42(6):3282. doi:10.1118/1.4924167.

[3] Bender ET, Hardcastle N, Tome WA (2012). On the dosimetric effect and reduction of inverse consistency and transitivity errors in deformable image registration for dose accumulation. Med Phys.

[4] Hardcastle N, Bender ET, Tome WA (2014). The effect on dose accumulation accuracy of inverse-consistency and transitivity error reduced deformation maps. Australas Phys Eng Sci Med.

[5] Martel MK, Ten Haken RK, Hazuka MB, Turrisi AT, Fraass BA, Lichter AS (1994). Dose-volume histogram and 3-D treatment planning evaluation of patients with pneumonitis. Int J Radiat Oncol Biol Phys.

[6] Sonke JJ, Belderbos J (2010). Adaptive radiotherapy for lung cancer. Semin Radiat Oncol.

[7] Graves YJ, Smith AA, McIlvena D, Manilay Z, Lai YK, Rice R, Mell L, Jia X, Jiang SB, Cervino L (2015). A deformable head and neck phantom with in-vivo dosimetry for adaptive radiotherapy quality assurance. Med Phys.

[8] Pukala J, Johnson PB, Shah AP, Langen KM, Bova FJ, Staton RJ, Manon RR, Kelly P, Meeks SL (2016). Benchmarking of five commercial deformable image registration algorithms for head and neck patients. J Appl Clin Med Phys.

[9] Park S, McNutt T, Plishker W, Quon H, Wong J, Shekhar R, Lee J (2016). Technical note: scuda: a software platform for cumulative dose assessment. Med Phys.

[10] Castadot P, Lee JA, Geets X, Gregoire V (2010). Adaptive radiotherapy of head and neck cancer. Semin Radiat Oncol.

[11] Wang W, Yang H, Hu W, Shan G, Ding W, Yu C, Wang B, Wang X, Xu Q (2010). Clinical study of the necessity of replanning before the 25th fraction during the course of intensity-modulated radiotherapy for patients with nasopharyngeal carcinoma. Int J Radiat Oncol Biol Phys.

[12] Yao WR, Xu SP, Liu B, Cao XT, Ren G, Du L, Zhou FG, Feng LC, Qu BL, Xie CB (2015). Replanning criteria and timing definition for parotid protection-based adaptive radiation therapy in nasopharyngeal carcinoma. Biomed Res Int.

[13] Brouwer CL, Steenbakkers RJ, Langendijk JA, Sijtsema NM (2015). Identifying patients who may benefit from adaptive radiotherapy: does the literature on anatomic and dosimetric changes in head and neck organs at risk during radiotherapy provide information to help?. Radiother Oncol.

[14] Dial C, Weiss E, Siebers JV, Hugo GD (2016). Benefits of adaptive radiation therapy in lung cancer as a function of replanning frequency. Med Phys.

[15] Guckenberger M, Richter A, Wilbert J, Flentje M, Partridge M (2011). Adaptive radiotherapy for locally advanced non-small-cell lung cancer does not underdose the microscopic disease and has the potential to increase tumor control. Int J Radiat Oncol Biol Phys.

[16] Weiss E, Fatyga M, Wu Y, Dogan N, Balik S, Wt S, Hugo G (2013). Dose escalation for locally advanced lung cancer using adaptive radiation therapy with simultaneous integrated volume-adapted boost. Int J Radiat Oncol Biol Phys.

[17] Thornqvist S, Hysing LB, Tuomikoski L, Vestergaard A, Tanderup K, Muren LP, Heijmen BJ (2016). Adaptive radiotherapy strategies for pelvic tumors - a systematic review of clinical implementations. Acta Oncol.

[18] Bratengeier K, Holubyev K (2016). Anisotropy of dose contributions-an instrument to upgrade real time IMRT and VMAT adaptation?. Med Phys.

[19] Bratengeier K, Oechsner M, Gainey M (2012). Methods for monitor-unit-preserving adaptation of intensity modulated arc therapy techniques to the daily target-a simple comparison. Med Phys.

[20] McCann C, Purdie T, Hope A, Bezjak A, Bissonnette JP (2013). Lung sparing and dose escalation in a robust-inspired IMRT planning method for lung radiotherapy that accounts for intrafraction motion. Med Phys.

[21] Sobotta B, Sohn M, Alber M (2010). Robust optimization based upon statistical theory. Med Phys.

